# Cancer of the Lips

**Published:** 1910-01-08

**Authors:** 


### CANCER OF THE LIPS.

Nowhere is the disproportion in the incidence of cancer in the two sexes better displayed than in the case of the lip. Critical examination of a large number of records shows cancer of the lip to be thirty times commoner in men than it is in women. Moreover, whereas in men one case in every fifteen of malignant disease occurs in the lip, in women the proportion is only one in seven hundred. It was formerly the custom to attribute this vast difference in sex incidence to the habit of smoking, but in view of the fact that many careful records exist of the occurrence of cancer of the lip in both men and women who have never either smoked or chewed tobacco, this explanation cannot reasonably receive support. It is best frankly to acknowledge that we do not know the reason for this any more than we know the cause of cancer; to throw overboard all the vague hypotheses and ill-substantiated speculations which enshroud the whole subject of cancer, and to confine our attention to ascertained facts. From the point of view of relative malignancy, cancer of the lip ranks low down in the scale. The duration of the disease is long, glandular infection is a late occurrence, and visceral metastasis is a very rare event.

Cancer of the lip is essentially a disease of old age. Mr. Jalland, of York, successfully removed a malignant growth from the lip of a man aged 103! The average age of all cases, however, is probably between fifty-five and sixty. It is interesting to recall the fact, often overlooked, that ten cases of a disease in men of sixty represents a much higher percentage incidence than a similar number of cases at thirty years of age, owing to the great decrease in the surviving population at the higher age. Just as striking as the disproportion in the sex incidence is the ratio in which the upper and lower lips are affected, for the lower lip is the seat of cancer thirty-five times more often than the upper. Satisfactory treatment of the disease when occupying the lower lip is a much simpler matter than when the upper lip is affected.

It is sometimes stated that cancer of the lip is of low malignancy, because operations for its removal are less frequently followed by recurrence. This is true in a sense, but only because the complete removal of the growth is a relatively easy matter. It is true that incomplete operations are just as certainly followed by recurrence in the case of the lip as they are in other situations of the body; and these recurrences are still far too numerous.

The operation which from the dawn of modern surgery has been almost universally practised is the simple V incision, accompanied or followed by excision of the lymphatic glands *when obviously enlarged.* It is a simple operation, it gives excellent cosmetic results, and would be entirely good did it furnish satisfactory results. One is bound to confess that it does not do this, and recently several attempts have been made to improve it. First, there is the question of the lymphatic glands. At the present time, in the case of the breast, no one thinks of leaving axillary lymphatic glands untouched because they cannot be felt to be enlarged, for pathological anatomy has taught us that no criterion short of microscopic examination will reveal the presence of a few epithelial cells in the sub-capsular lymph space of a lymphatic gland. The operation for cancer of the breast must be regarded, in our present state of knowledge, as a type operation, and all other operations for malignant disease should be planned on this.

Further, a little thought on the anatomy of the lip and the arrangement of its lymphatics should teach us that the growth, rather than travel downwards within the confines of the narrow V, must almost certainly extend in a fan-shaped manner, the growth forming the apex of the fan rather than its base. In these circumstances it would appear more reasonable so to place the incision that a quadrilateral area of the lip is removed, the lower edge of the quadrangle being considerably larger than the upper which includes the growth. From the lower end of the quadrangle another incision should be carried over the chin and along the corresponding submaxillary triangle; and then the quadrilateral portion of lip, the submental and submaxillary lymphatic area and that portion of the fasciæ connecting the two can be dissected out in a continuous sheet. By undercutting and sliding flaps, a cosmetic result almost as good as that given by the old operation should be obtained and certainly a much greater chance of freedom from either local or glandular recurrence.

